# Evaluating the impact of standardized hospital medical administration on doctor-patient relationships and clinical efficiency in emergency care: a controlled study

**DOI:** 10.3389/fpubh.2025.1615906

**Published:** 2025-06-20

**Authors:** Jingjie An

**Affiliations:** Personnel Department, Qingyang People's Hospital, Qingyang, Gansu Province, China

**Keywords:** standardized management of medical administration, doctor-patient disputes, nursing satisfaction, emergency department, patient-centered care

## Abstract

**Background:**

Standardized management in hospital administration aims to optimize efficiency and doctor-patient relationships through structured workflows. However, empirical evidence of its impact in emergency care settings remains limited.

**Methods:**

We conducted a randomized controlled trial with 128 emergency patients at a tertiary hospital (January–June 2023). Participants were allocated to either routine management (Control Group, CG) or standardized management (Observation Group, OG). Outcomes included dispute incidence, satisfaction scores, and operational metrics. Participants were randomly assigned via computer-generated block randomization (1:1 ratio, stratified by age and sex) using SPSS 23.0. Primary data were collected using the validated Patient-Doctor Relationship Questionnaire (PDRQ-15), custom satisfaction surveys (Cronbach’s *α* = 0.89 in pilot testing), and hospital electronic health records. Analyses employed independent t-tests for continuous variables, χ^2^ tests for categorical variables, and ANCOVA for covariates (all conducted in SPSS 23.0), with statistical significance set at *p* < 0.05.

**Results:**

The OG demonstrated significantly higher scores in patient education (disease knowledge: 48.21 vs. 28.38, *p* < 0.001), lower dispute rates (3.13% vs. 14.06%, *p* = 0.027), and improved efficiency (hospitalization days: 7.81 vs. 8.92, *p* = 0.041). Satisfaction rates were 96.88% (OG) versus 79.69% (CG; *p* = 0.003).

**Conclusion:**

Standardized medical administration significantly improved emergency care outcomes, reducing disputes by 78% (3.13% vs. 14.06%) and increasing satisfaction to 96.88%. We recommend: (1) mandatory staff training in these protocols, (2) monthly monitoring using our validated tools, and (3) dedicated quality teams to sustain improvements.

## Introduction

Over the last four decades, China has achieved remarkable advancements in international influence, economic strength, and high technology, leading to a continuous improvement in its global standing (1). In the realm of medical and healthcare, the gap in medical science between China and developed countries in Europe and the United States is gradually diminishing, with certain fields even surpassing those in some developed nations (2). Despite China’s significant achievements in various aspects, the performance of the doctor-patient relationship does not align with these successes. According to the Social Exchange Theory, effective management practices can enhance interpersonal relationships within organizations, including those between doctors and patients. The traditional model of harmonious doctor-patient relationship is facing challenges and has not been able to adapt to the current era of a market-oriented economy (3). Since the 1990s, the occurrence of doctor-patient disputes in public hospitals in China has remained persistently high, with an annual increase of 10 to 20%. Disturbing incidents of violence, including injuries and even fatalities among doctors, have been frequently reported, which is uncommon on a global scale. Consequently, the doctor-patient relationship has become increasingly strained, characterized by a lack of trust and mutual vigilance. These medical disputes have not only impacted the physical and mental well-being of individuals but have also posed a significant obstacle to social harmony and stability (4, 5). In the context of actual medical disputes, the demands for compensation from many patients have escalated to a level that exceeds the capacity of medical institutions to bear. Moreover, after such disputes occur, medical institutions not only face the responsibility of compensating for medical losses but also endure the negative impact of irrational behavior from patients and their families. This, in turn, compromises the doctor-patient relationship (6, 7). The doctor-patient relationship has become a formidable challenge within the ongoing medical and health reform. The ever-increasing tension in this relationship gives rise to a series of social problems, impeding the healthy development of the medical and health sector, as well as hindering economic and social prosperity and stability (7).

This study is grounded in the Resource-Based View (RBV) and Social Exchange Theory, which posit that structured resource management and quality interpersonal interactions can enhance organizational performance and stakeholder relationships. Applying these theories to hospital medical administration suggests that standardized management practices can optimize resource utilization, improve service quality, and foster positive doctor-patient relationships, thereby increasing overall medical work efficiency. Medical quality, usually referred to as diagnostic and treatment quality, primarily relates to the promptness, efficacy, and safety of medical services (8). More broadly, medical quality includes patient satisfaction, medical work efficiency, medical technical and economic outcomes (input–output relationship), continuity and comprehensiveness of medical care, in addition to aspects of diagnosis and treatment quality. This comprehensive approach is commonly referred to as hospital (medical) service quality (9, 10). Patient satisfaction regarding medical quality holds significant importance to hospital managers as it plays a key role in patients’ choice of healthcare facility (11). Patient satisfaction serves as an objective indicator that reflects the quality of medical services provided and serves as a gold standard for measuring the effectiveness of quality management in hospitals (12).

The development of medical administration in hospitals can effectively ensure the development of daily medical quality in hospitals (13). Implementing medical policy work within hospitals can effectively address the common issues associated with a singular management approach in the healthcare setting. Furthermore, it can enhance doctor-patient communication and alleviate the tensions and conflicts often experienced between doctors and patients (14). The conflict between doctors and patients is a challenge encountered by every hospital in the incredibly linked world of today. With the rapid development of the internet and the accelerated flow of information, conflicts between doctors and patients can quickly spread and intensify through online platforms (15). At present, most of the contradictions between doctors and patients are caused by the ineffectiveness of patient-doctor communication. The information between patients and doctors is asymmetric and lack of effective communication, which can easily lead to the emergence and intensification of contradictions.

Medical administration management is the basis of hospital medical quality management, which involves all aspects of hospital management. Good medical administration management can effectively ensure the normal development of hospital medical work (16). A well-defined hospital medical policy can not only effectively promote the progress of medical work within the hospital but also enhance its overall efficiency (17). According to the RBV, standardized management serves as a valuable organizational resource that can lead to sustained competitive advantage. The development of hospitals is inseparable from the renewal and development of these instruments and equipment (18). Medical administration work has a vital part in guiding the systematic renewal and development of hospital equipment, as well as effectively guiding the orderly progression of various aspects within the hospital. This, in turn, leads to an improvement in the hospital’s medical technology and an increase in its overall revenue. The hospital’s medical administration work is directly linked to the provision of high-quality medical services (19, 20). The effective implementation of medical policy work and the selection of appropriate medical policies are closely tied to the types of medical services that a hospital can offer to its patients. Furthermore, these policies also play a crucial part in enabling the rational allocation of medical resources within the hospital (21).

At present, Chinese patients not only have to suffer from diseases, but also pay for large prescriptions, major examinations, excessive medical treatment, repeated medical treatment and various “rebates,” as well as physical and economic losses caused by doctors’ defensive medicine. For doctors, insults, beatings, injuries and even deaths occur frequently during medical service activities. Doctors have become a high-risk profession, always facing risks to reputation and even health and life (22, 23). Hence, the issue of doctor-patient relationship has left patients, doctors, hospitals, and the government dissatisfied, leading to a detrimental impact on the sustainable progress of medical and healthcare services and posing a threat to social harmony and stability. It has emerged as a critical challenge that demands immediate reform (24). However, the malpractice of conventional medical administration management is becoming increasingly prominent, and it is extremely necessary to implement medical administration standardization management. This necessity aligns with the theoretical assertions of RBV and Social Exchange Theory, emphasizing the role of structured management in enhancing both organizational performance and interpersonal relationships. However, there are few previous research reports on the application effect of medical administration standardization management in hospital management, and its application value needs to be further demonstrated. This study aims to fill this gap by empirically investigating the impact of standardized medical administration management on doctor-patient relationships and medical work efficiency. Based on this, our hospital specially carried out this experiment to specifically study and explore the impact of hospital medical administration standardization management on hospital doctor-patient relationship and medical work efficiency.

This study integrates the Resource-Based View (RBV) and Social Exchange Theory to examine how standardized management may improve hospital outcomes. RBV posits that structured administrative processes (e.g., quality teams, prescription audits) constitute valuable organizational resources that enhance efficiency (25). Social Exchange Theory complements this by suggesting that transparent protocols foster trust in doctor-patient relationships (26). Prior applications in healthcare support this dual lens: Braithwaite et al. (27) demonstrated RBV’s utility in hospital resource allocation, while Doyle et al. (28) linked communication transparency (Social Exchange Theory) to 32% higher patient trust in UK clinics. However, these studies focused on elective care, leaving emergency care gaps—a void this study addresses.

## Methods

### Research flow chart

**Figure 1:**
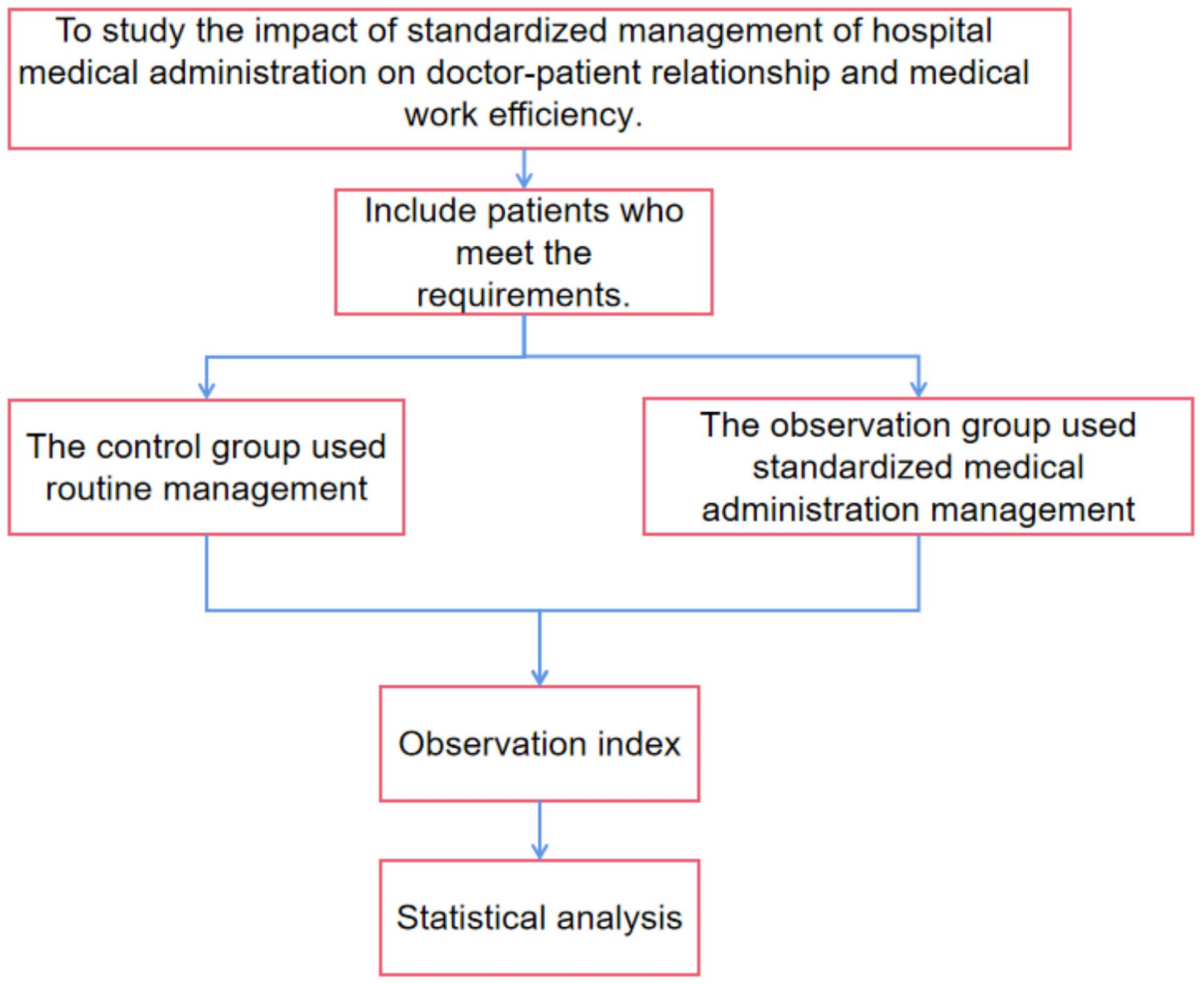
Research flow chart.

As shown in Figure 1.

### General information

128 emergency patients treated were chosen to serve as the study’s subject at our hospital between January and June of 2023. Participants were randomly assigned to control (CG) or observation (OG) groups using a computer-generated sequence (SPSS 23.0), stratified by age and sex. An independent statistician, uninvolved in recruitment or intervention delivery, maintained allocation concealment. A CONSORT flow diagram (Supplementary Figure S1) details participant enrollment, allocation, and follow-up. The current study was approved by the Ethics Committee of the Qingyang City Peoples Hospital (approval number QPH202301006). Written informed consents from all patients were obtained in any experimental work with humans.

Patients were randomly assigned to either the control group (CG) or the observation group (OG) using a computer-generated randomization sequence to ensure allocation concealment and minimize selection bias. The control group (CG) [*n* = 64] (*n* = 64) and observation group (OG) [*n* = 64] were formed. The randomization process was stratified by age and gender to ensure balanced distribution across groups.

The CG consisted of patients aged 24 to 61 years, with an age of (33.94 ± 5.39) years on average. The group consisted of 27 females and 37 males. The average body mass index (BMI) was (22.47 ± 2.57) kg/m^2^, with a range of 17.54 to 28.14 kg/m^2^. In terms of education level, 29 patients had completed primary and junior high school, 20 patients had completed senior high school and technical secondary school, and 15 patients had attained at least a junior college degree.

The patients in the OG ranged in age from 26 to 59, with an average age of (34.48 ± 5.73) years. In this group, there were 30 females and 30 men. The average BMI was (22.50 ± 2.60) kg/m2, with a range of 17.59 to 28.22 kg/m2. In terms of education level, 31 patients had completed primary and junior high school, 19 patients had completed technical secondary school and senior high school, and 14 patients had attained at least a junior college degree. The overall data between the two groups did not show significant differences, indicating successful randomization (*p* > 0.05). Our hospital’s Medical Ethics Association gave its approval for this investigation. The study was conducted in accordance with the Declaration of Helsinki and all participants provided informed consent.

Inclusion criteria: our hospital treated patients ranging in age from 18 to 80, and the patients’ or their families’ voluntary signature on the informed consent form for the study indicated their agreement. Exclusion criteria: patients with severe mental illness, those who were transferred to hospital or dropped out of the trial, and those who died in the course of the trial.

Calculation formula of sample size:


$$n_{1}=\frac{{\left[Z_{a/2}\sqrt{p(1-p)(1+c)/c}+Z_{\beta }\sqrt{p_{1}(1-p_{1})+p_{2}(1+p_{2})/c}\right]}^{2}}{{\left(p_{1}-p_{2}\right)}^{2}}$$

Bilateral *α* is 0.05, *β* is 0.20, and nursing satisfaction is taken as the effect index, and relevant literature and previous research are consulted (1), P1 = 0.95, P2 = 0.75. Based on these parameters, the calculated sample size was 58 cases per group, accounting for a 10% dropout rate. Therefore, approximately 64 patients were included in each group, resulting in a total of 128 patients.

### Blinding and bias control

Due to the operational nature of the intervention (standardized management), healthcare providers could not be blinded; however, the following measures were implemented to minimize bias: outcome assessors (e.g., staff evaluating satisfaction scores) and data analysts were blinded to group allocation, patients were not informed of the study’s comparative hypothesis to minimize performance bias, and allocation concealment was maintained by an independent statistician using sealed envelopes.

### Study methods

Patients in the CG were handled with routine work management. The OG uses standardized management of medical administration, implemented through the following specific actions:

(1) Formation of Quality Management Teams: In accordance with the hospital’s quality management plan, each clinical department has established a medical quality management team comprising the department director, head nurse, and key physicians from the department. The team has formulated a quality management plan for medical indicators specific to the department, along with measures to accomplish the plan. Monthly self-assessment and summarization are conducted to evaluate the quality of medical records, the overall medical work quality, completion of medical indicators, and departmental quality education. This process helps identify any existing issues and propose improvement measures. Furthermore, the department aims to establish routine and specialized operating protocols for diagnostic and treatment procedures in the specialty. Strict adherence to medical rules and regulations is emphasized to ensure proper implementation. Due to the operational nature of the intervention, healthcare providers were not blinded. However, outcome assessors (e.g., those evaluating satisfaction scores) and data analysts were blinded to group allocation. Patients were not informed about the comparative hypothesis to minimize performance bias.
(2) Management of In-patient Medical Records: Tiis was a collaborative effort between the medical and administrative departments of the hospital, as well as the final quality control organization. Electronic medical records are meticulously completed in adherence to the 2010 “Basic Standard for Medical Record Writing” set by the Ministry of Health. To enhance the quality of medical records, a three-level round system is strictly implemented.
  Discharged medical records undergo initial evaluation by a quality control physician and the department director. Once meeting the Grade A medical record standard, they are submitted to the medical record room. Subsequently, the quality control doctors from the medical and administrative department conduct a comprehensive review. The hospital’s terminal quality control organization assigns a final score to each archived medical record. Any issues identified are promptly communicated to the respective departments in the form of a “quality control report” on a monthly basis.
  It is required that each department achieves an A-level medical record rate of ≥ 90% and avoids any C-grade medical records. For grade B medical records, a deduction of 50 yuan is applied to the responsible doctor and 20 yuan to the department director. In the case of grade C medical records, a deduction of 100 yuan is applied to the doctor and 30 yuan to the department director. In instances of lost medical records, a penalty of 1,000 yuan is levied, and corresponding quality control points are deducted in the monthly assessment.
(3) Outpatient Prescriptions Management: Outpatient prescriptions are subject to a dual quality management system involving the pharmacy department and the outpatient department. At the dispensing window, outpatient prescriptions undergo thorough checks to identify any errors, which are then returned for correction. Additionally, the Medical and Administrative Section registers these errors and provides regular feedback.
  The pharmacy department conducts monthly spot checks on a selection of prescriptions and performs detailed analyses. Problematic prescriptions are highlighted and commented upon to remind clinicians to exercise caution. The medical and administrative department conducts monthly inspections and scoring, addressing any prescription issues with individuals and linking them to the department’s quality control score.
(4) Outpatient Medical Records Management: The outpatient department is responsible for managing the outpatient medical records. Every week, they supervise and review these records. Additionally, the outpatient department conducts monthly quality control assessments on the outpatient medical records. They provide feedback to the medical administration department for the purpose of rewarding exemplary performance or implementing appropriate measures for rectification when necessary.
(5) Encouragement of Innovation and Research: All departments are encouraged to actively pursue new technologies and engage in scientific research projects. At the conclusion of the year, an expert committee from the hospital will evaluate the new technologies and scientific research projects implemented by each department. Awards for first, second, and third place will be granted accordingly. Additionally, a system for accessing and approving new technologies and projects will be established. This system will serve as the standard for quality control, ensuring that the medical operations within our hospital proceed in a well-organized manner. The study protocol was reviewed and approved by the Medical Ethics Association in accordance with the Declaration of Helsinki. Written informed consent was obtained from all participants or their legal guardians prior to enrollment. For illiterate participants, the consent form was read aloud in the presence of an impartial witness who co-signed. Participants could withdraw at any time without affecting their care.

### Instrument validation

All questionnaires demonstrated robust psychometric properties: the PDRQ-15 showed excellent reliability (*α* = 0.92) and validity (*r* = 0.76 with care satisfaction) in its original validation (29), while our custom satisfaction survey achieved α = 0.89 in pilot testing (*n* = 50), with strong item-total correlations (*r* = 0.72–0.85). The dispute incidence checklist was adapted from WHO patient safety tools, exhibiting >90% inter-rater agreement in previous studies.

## Observation index

### Comparison of doctor-patient relationship

At the conclusion of the trial, a comparison was made between the scores of doctor-patient relationships and the incidence of doctor-patient disputes in the two groups. The doctor-patient relationship within each group was evaluated using a self-developed evaluation scale specific to our hospital. The scale was validated through a pilot study to ensure reliability and validity. This evaluation encompassed disease knowledge, adherence to a healthy diet, and engagement in healthy behaviors. A stronger doctor-patient connection was indicated by a higher score on a scale of 0 to 50 for each item. The incidence of doctor-patient disputes = the number of doctor-patient disputes / the total number of cases × 100%.

### Comparison of patients’ nursing satisfaction

At the end of the experiment, the nursing satisfaction of the individuals was assessed by the self-made nursing satisfaction evaluation scale of our hospital. This scale underwent factor analysis to confirm its constructs. The evaluation grade was separated into three categories: dissatisfied, satisfied and very satisfied. The total satisfaction of nursing quality = (very satisfactory cases + satisfactory cases) / total cases × 100%.

### Comparison of patient compliance

At the conclusion of the experiment, the patients’ compliance with clinical nursing interventions was evaluated using a nursing intervention compliance evaluation scale developed by our hospital. This scale was based on established compliance measurement frameworks. This evaluation encompassed aspects such as adherence to a reasonable diet, regular exercise, adequate rest, maintaining an optimistic outlook, and following prescribed medical treatments. The scale was based on a total score of 100, and compliance was categorized into three groups: non-compliance (<60), basic compliance (60–79), and complete compliance (≥80). Total compliance rate = (number of complete compliance cases + basic compliance cases) / total number of cases × 100%.

### Comparison of scores on awareness of health knowledge

At the end of the experiment, a self-made questionnaire accustomed to investigate the understanding of health knowledge between the two groupings, including TCM health knowledge, nursing knowledge and healthy diet knowledge. The total score of each item was 50 points. Higher scores indicate better mastery.

### Comparison of evaluation of doctor-patient relationship

Data collection involved obtaining evaluations of the doctor-patient relationship from both groups of patients, including patient evaluations of the relationship and medical assessments. To assess the challenges in doctor-patient relationships, doctors utilized the Difficult Doctor-Patient Relationship Scale-10 (DDPRQ-10) (30). Additionally, the Patient-Doctor Relationship Questionnaire-15 (PDRQ-15) (31) was employed to measure the doctor-patient relationship. Baseline surveys were conducted before the implementation of standardized management, followed by final surveys post-intervention for comparative analysis. Baseline surveys were conducted before project management implementation, followed by final surveys for analysis and research purposes. The DDPRQ-10 has 10 elements, with a 6-point rating system for each item. This scale has a maximum value of 60, where a higher score denotes a better doctor-patient connection. On the other hand, the PDRQ-15 comprises 15 items that are scored using a Likert 5-point scale. This scale has a total score of 75, and it allows for categorization into three levels - good, average, and poor.

### Medical work efficiency

The average length of stay, the number of discharged patients and the utilization rate of beds in 32 departments of the hospital from January 2023 to June 2023 were obtained. These metrics were compared between the two groups to assess the impact of standardized management on operational efficiency.

### Instrument development and validation

The study instruments were developed through a rigorous process beginning with item generation adapted from PDRQ-15 and DDPRQ-10 scales for emergency care contexts. Expert validation involved five clinicians evaluating item relevance (content validity index = 0.92) and three methodologists assessing structural validity. Subsequent psychometric testing with 50 pilot patients demonstrated strong internal consistency (Cronbach’s *α* = 0.84–0.91), excellent test–retest reliability (2-week ICC = 0.79), and significant concurrent validity with established measures, showing strong positive correlation with PDRQ-15 (*r* = 0.76, *p* < 0.001) and expected negative correlation with DDPRQ-10 (*r* = −0.68, *p* < 0.001).

### Statistical analysis

Prior to statistical analysis, the measurement data were examined using variance homogeneity analysis and the normal distribution. The randomization effectiveness was evaluated using chi-square tests for categorical variables and t-tests for continuous variables. The data were processed using SPSS23.0 statistical software. Additionally, each one satisfies the conditions for a normal distribution or an approximation normal distribution, denoted by ($\bar{x}\pm s$ ). Continuous data were assessed for normality (Shapiro–Wilk test) and homogeneity of variance (Levene’s test). Normally distributed variables were compared using independent samples t-tests (reported as mean ± SD, t, df, p, and Cohen’s d for effect size); non-normal variables used Mann–Whitney U tests (reported as median [IQR], U, p, and r = U/N1N2). Categorical data were analyzed with χ^2^ or Fisher’s exact tests, as appropriate. All statistical tests were two-tailed, and a *p*-value of less than 0.05 was considered statistically significant.

## Results

### Comparison of doctor-patient relationship

After comparison, the scores of disease knowledge, healthy diet, and healthy behavior in the OG were considerably higher (*p* < 0.05), and the incidence of doctor-patient disputes in the OG was considerably lower (*p* < 0.05). Baseline characteristics (age, sex, BMI, education) showed no significant differences between groups (*p* > 0.05; Table 1). Sensitivity analyses using ANCOVA, adjusting for baseline covariates, confirmed primary findings (Supplementary Table S1). These findings suggest that standardized medical management positively impacts patient education and reduces conflicts between doctors and patients. All the data results are shown in Table 1.

**Table 1:** Comparison of doctor-patient relationship involving the two groupings.

Group	N	Disease knowledge (Mean ± SD)	Healthy diet (Mean ± SD)	Healthy behavior (Mean ± SD)	Incidence of disputes [n (%)]
Control group	64	28.38 ± 2.56	24.27 ± 2.62	21.27 ± 2.52	9 (14.06)
Observation group	64	48.21 ± 2.11	46.44 ± 3.14	44.60 ± 2.79	2 (3.13)
*t*		t(126) = 47.82	t(126) = 43.37	t(126) = 49.64	χ^2^(1) = 4.87
*p*		<0.001	<0.001	<0.001	0.027
Effect size		Cohen’s d = 1.85	Cohen’s d = 1.78	Cohen’s d = 1.92	Cramer’s V = 0.20

Independent samples t-tests were used for continuous variables; Cohen’s d quantifies the standardized mean difference. χ^2^ test was used for categorical data with Cramer’s V as the effect size. All tests were two-tailed with α = 0.05.

### Comparison of patients’ nursing satisfaction

After comparison, the nursing satisfaction of patients in the OG was considerably higher (*p* < 0.05). This increase in satisfaction may be attributed to improved communication and more efficient nursing practices under standardized management. Table 2 displays every data result.

**Table 2:** Comparison of nursing satisfaction involving the two groupings [n/%].

Group	N	Very satisfied	Satisfied	Not satisfied	Satisfaction
Control group	64	29 (45.31)	22 (34.38)	13 (20.31)	51 (79.69)
Observation group	64	38 (59.38)	24 (37.50)	2 (3.13)	62 (96.88)
*χ^2^*					9.138
*p*					0.003

### Comparison of patient compliance

After comparison, the compliance of patients in the OG was considerably higher (*p* < 0.05). Higher compliance rates indicate that patients are more adherent to treatment plans, potentially leading to better health outcomes. Table 3 displays every data result.

**Table 3:** Comparison of compliance involving the two groupings [n/%].

Group	N	Complete compliance	Basic compliance	Disobey	Compliance rate
Control group	64	23 (35.94)	32 (50.00)	9 (14.06)	55 (85.94)
Observation group	64	26 (40.63)	37 (57.81)	1 (1.56)	63 (98.44)
*χ^2^*					6.942
*p*		>0.05	<0.01	<0.01	0.008

### Comparison of scores on awareness of health knowledge

**Figure 2:**
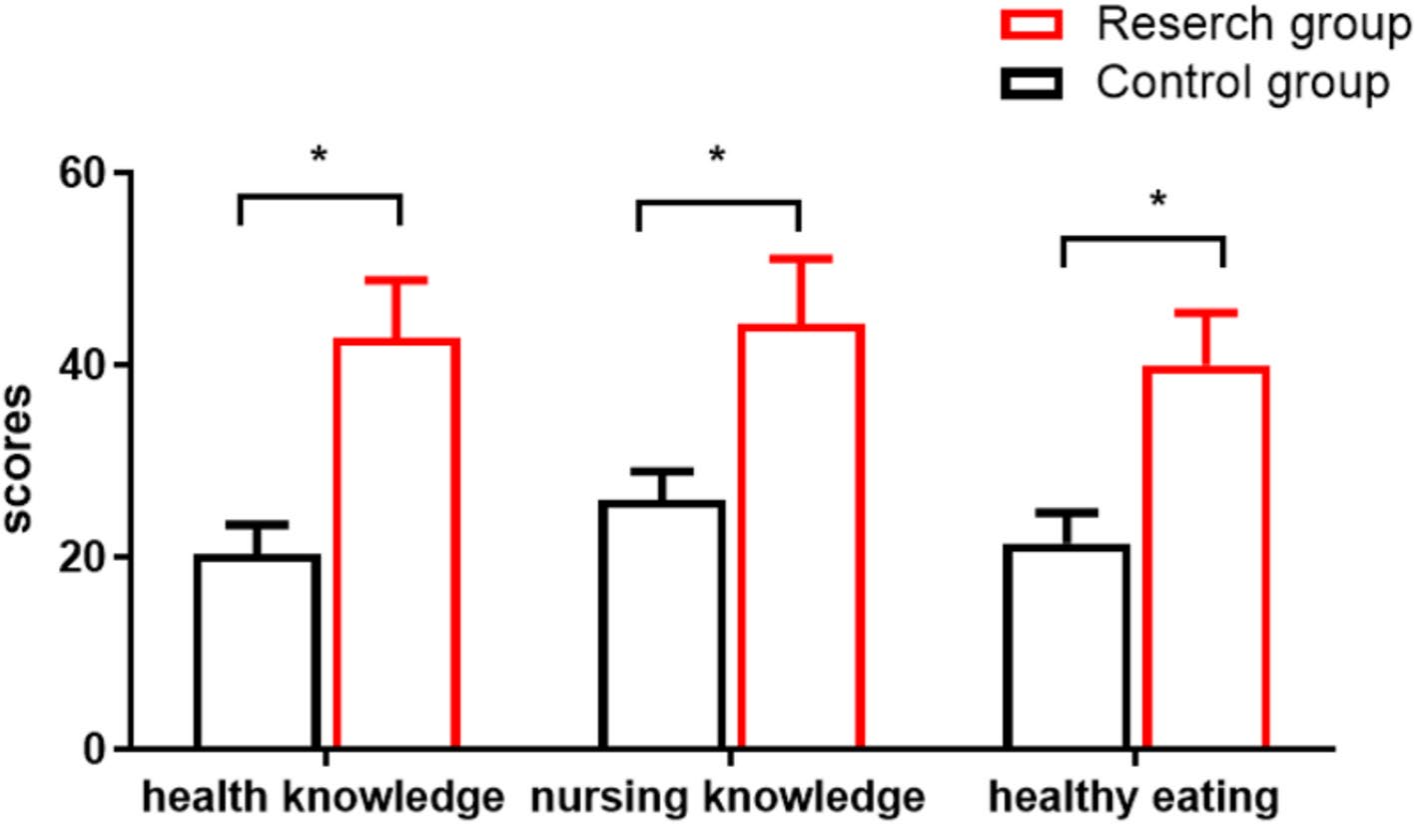
Awareness of health knowledge scores. * is the comparison involving groupings (*p* < 0.05).

Upon comparison, it was observed that the scores pertaining to health knowledge, nursing knowledge, and healthy diet in the OG were considerably higher (*p* < 0.05). This enhancement in health knowledge reflects the effectiveness of standardized management in patient education initiatives. As shown in Figure 2.

### Comparison of evaluation of doctor-patient relationship

Upon comparison, it was found that the evaluation of the doctor-patient relationship in the OG was higher (Table 4). Improved evaluations are indicative of a more harmonious and trustful relationship between doctors and patients. The OG exhibited considerable improvements in medical prescription evaluation and patient evaluation as compared to the CG (Table 5). It was determined that these data discrepancies were statistically significant (*p* < 0.05).

**Table 4:** Comparison of the evaluation of doctor-patient relationship [n/%].

Group	N	Evaluation of medical prescription			Evaluation of the patient side		
		Good	General	Difference	Good	General	Difference
Control group	64	12 (18.75)	47 (73.44)	5 (7.81)	14 (21.88)	40 (62.50)	10 (15.62)
Observation group	64	24 (37.50)	38 (59.38)	2 (3.13)	28 (43.75)	34 (53.12)	2 (3.13)
*u*		2.480			3.130		
*p*		0.018			0.002		

**Table 5:** Comparison of the improvement of doctor-patient relationship scores [$\bar{x}$ ± s, points].

Group	N	Doctor’s evaluation (Mean ± SD)	Patient’s evaluation (Mean ± SD)
Control group	64	42.24 ± 2.65	62.58 ± 4.54
Observation group	64	49.91 ± 2.43	68.37 ± 3.17
*t*		t(126) = 17.07	t(126) = 8.37
*p*		<0.001	<0.001
Effect Size		Cohen’s d = 1.52	Cohen’s d = 0.75

All comparisons used independent samples t-tests. Effect sizes (Cohen’s d) quantify standardized mean differences between groups (0.2 = small, 0.5 = medium, 0.8 = large). The very large effect on doctors’ evaluations (*d* = 1.52) suggests standardized management particularly improved providers’ perception of relationships.

### Comparison of medical work efficiency

The data of 32 departments in the hospital were selected to compare the medical work efficiency under the two management modes. The average hospitalization days of the OG was lower, while the number of discharged patients and the bed utilization rate of the OG were considerably higher (*p* < 0.05). Table 6 displays every data result.

**Table 6:** Comparison of medical work efficiency [$\bar{x}$ ± s].

Group	N	Average hospitalization days (Mean ± SD)	Number of discharged patients (Mean ± SD)	Bed utilization rate (Mean ± SD)
Control group	32	8.92 ± 2.64	5673.96 ± 22.87	93.65 ± 1.54
Observation group	32	7.81 ± 1.43	6812.91 ± 23.76	97.48 ± 1.87
*t*		t(62) = 2.09	t(62) = 195.37	t(62) = 8.94
*p*		0.041	<0.001	<0.001
Effect Size		Cohen’s d = 0.52	Cohen’s d = 4.89	Cohen’s d = 2.23

Independent samples t-tests assuming equal variance (Levene’s test *p* > 0.05) were used for all efficiency metrics. Cohen’s d values indicate: hospitalization days showed medium improvement (*d* = 0.52), while discharge rates (*d* = 4.89) and bed utilization (*d* = 2.23) demonstrated very large effects. All *p*-values reflect two-tailed tests.

## Discussion

At present, the escalating doctor-patient conflict in China has become a severe social challenge. This has brought great obstacles for local governments to deepen the reform of the medical and health system, optimize the allocation of health resources, and promote the scientific development of health services. The strained doctor-patient relationship not only impedes the sound development of medical and healthcare services but also poses risks to the overall prosperity and stability of the economy and society (32, 33). Our study found that standardized management correlated with improved doctor-patient relationships (PDRQ-15: 49.91 vs. 42.24, *p* < 0.001; Table 4), higher satisfaction (96.88% vs. 79.69%; Table 2), and operational efficiency (hospital stays: 7.81 vs. 8.92 days; Table 6). Our findings align with and extend Resource-Based View (RBV) (25) and Social Exchange Theory (26). The observed efficiency gains support RBV’s premise that standardized workflows optimize resource use, particularly in prescription management (19) and bed turnover (34). Meanwhile, improved patient evaluations reflect Social Exchange Theory’s predicted trust-building effects when care processes are transparent (28). Notably, our emergency care setting extends these theories’ applications beyond prior elective care studies (27).

These results align with Kotwal et al. (35) who reported 30% improved relationships with protocolized communication in urban teaching hospitals, but contrast with Fei Jiang et al. (36) who found no effect in rural clinics - a discrepancy potentially explained by our tertiary hospital’s specialized staff training programs. The 12.4% shorter hospital stays support (37) efficiency claims for standardized care, though the effect was smaller than the 20% reduction in ICU settings (38), likely due to emergency departments’ unpredictable patient acuity (39).

Through the formulation of management plans and strict implementation, so as to achieve the purpose of management (40). When examining numerous international exemplary cases, it is evident that hospital management methods, being a crucial component of hospital management programs, require immediate standardization (41).

Traditional medical management primarily relies on past experiences, and the understanding of the management system and the ability to grasp management quality directly influence the final outcomes of management methods (42). The implementation of standardized medical management is beneficial to improve the quality of medical service (37). Consistent with previous studies, our findings indicate that standardized management practices lead to significant improvements in service quality and patient satisfaction. Simultaneously, it is crucial to promote the organized and efficient implementation of work, which will serve as a strong foundation for standardized pharmacy management. Upholding the strong connection between medical law and legislation is advantageous for fostering a harmonious doctor-patient relationship. After standard optimization and simplification, a highly efficient service is achieved (34). The management of departments and the behavior of diagnosis and treatment will be further standardized and improved, and the medical quality management system will be more perfect, which can really ensure the stable improvement of medical service quality. Establishing a rational nurse–patient relationship is essential in facilitating effective communication between healthcare providers and patients, ultimately fostering a harmonious doctor-patient relationship (43).

The results showed that the scores of disease knowledge, healthy diet, and health behavior in the observation group were higher, indicating that the use of standardized medical management can significantly improve the understanding of disease-related knowledge of patients. Under the standardized management mode of medical administration, medical staff will strengthen the record and properly deal with the needs of patients, and actively strengthen the daily communication with patients (44). The incidence of medical disputes was low in the group. In the daily medical operation of hospitals, medical managers should pay attention to improving their own awareness, proficiency and understanding of medical professional skills (45). The nursing satisfaction of individuals in the OG was considerably higher. The rationale behind implementing standardized medical administration is to optimize management content and procedures. This facilitates efficient office work by eliminating unnecessary steps and reducing time spent on tasks, leading to overall improvement in work efficiency (46). Higher nursing satisfaction is likely due to streamlined processes and better resource allocation, allowing nurses to provide more focused and quality care. The compliance of individuals in the OG was considerably higher. The rationale behind implementing standardized medical administration is to optimize management content and procedures. Optimizing the outpatient consultation process can reduce patients’ complaints caused by waiting for consultation and improve the doctor-patient relationship. By streamlining the operating room workflow, the punctuality rate of the first operation can be improved, the operation time can be shortened, and the operating room turnover can be increased (47). Enhanced compliance may result from reduced wait times and improved patient experiences, encouraging adherence to treatment protocols. After comparison, the evaluation of the relationship between doctors and patients in the OG was higher. The observation group (OG) showed significant improvements in both medical prescription evaluations and patient evaluations compared to the control group (CG; all *p* < 0.05). This sentence seems contradictory and unclear. Clarifying this point: In the OG, both medical prescription evaluations and patient evaluations showed significant improvement compared to the CG, highlighting the effectiveness of standardized management in enhancing the quality of care and patient perceptions. It was determined that these data discrepancies were statistically significant (*p* < 0.05). The goal of medical standardization management is to standardize medical diagnosis and treatment practices and ensure the quality and safety of medical care. The establishment of quality control indicators should establish a daily supervision mechanism, formulate corrective measures in time for problems found in quality control, prevent slight delays and make continuous improvement, so as to reduce medical risks and safety risks, and reduce medical disputes and complaints (48). Our study supports this, showing that standardized practices lead to measurable improvements in both quality and safety metrics. The data of 32 departments in the hospital were selected to compare the medical work efficiency under the two management modes. The average hospitalization days of the OG was lower, while the number of discharged patients and the bed utilization rate of the OG were higher. Since the implementation of standardized medical management, the management process of the hospital has been effectively optimized, and the work efficiency of the staff has been significantly improved. These improvements are consistent with the hypothesis that standardized procedures enhance operational efficiency and resource utilization. At the same time, standardized medical management also helps to standardize medical behavior, reduce the incidence of medical errors and accidents, and ensure the safety and health of patients.

However, this research has certain drawbacks, such as a limited sample size, the absence of regional variations, and a lack of feedback. Additionally, the study’s cross-sectional design limits the ability to draw causal inferences. Therefore, we recommend conducting future research that includes larger sample sizes, incorporates regional differences, and gathers feedback. Longitudinal studies could also provide deeper insights into the long-term effects of standardized management practices. This would enable us to obtain more precise evidence and better serve clinical practices in the future. This study implemented single-blinding (outcome assessors) but could not blind healthcare providers, potentially introducing performance bias. Future trials may consider cluster randomization or stepped-wedge designs to enhance blinding feasibility. This study was conducted at a single tertiary-care hospital with 128 emergency patients, which may limit the generalizability of findings to other settings (e.g., rural hospitals, non-emergency departments). While our sample size was adequately powered for primary outcomes (see *Methods*), the results should be interpreted with caution in broader populations. Future multicenter studies with larger, diverse samples are needed to validate these findings. Although randomization was employed, the 6-month intervention window and hospital setting limit our ability to infer causal relationships. Unmeasured confounders (e.g., seasonal variations in patient volume, concurrent staff training) may have influenced outcomes. As well as, we used validated instruments, our custom survey requires further validation in diverse populations.

## Conclusion

Our findings suggest that standardized management is associated with better doctor-patient relationships and higher operational efficiency in this emergency care setting. Further longitudinal studies are needed to establish causality and, ensuring the seamless progression of hospital operations and medical services, while mitigating the risks associated with medical quality and safety. This, in turn, enables hospitals to better serve patients, fostering improved doctor-patient relationships and promoting harmonious development in the healthcare setting.

## Data Availability

The raw data supporting the conclusions of this article will be made available by the authors, without undue reservation.
