# Supplementary material for: Evaluating the impact of standardized hospital medical administration on doctor-patient relationships and clinical efficiency in emergency care: a controlled study
Source: Front Public Health. 2025 Jun 20;13:1615906. doi: 10.3389/fpubh.2025.1615906 (PMC12226502; doi:10.3389/fpubh.2025.1615906)
Supplement: Supplementary file 1 [file Supplementary_file_1.docx]

**Supplementary Table S1**

**ANCOVA Results for Primary Outcomes Adjusted for Baseline Covariates (Age, Sex, BMI, Education)**

| **Outcome Measure** | **Group** | **Unadjusted Mean ± SD** | **Adjusted Mean (95% CI)*** | ***P*-value (Adjusted)** | **Effect Size (η²)** |
| --- | --- | --- | --- | --- | --- |
| Disease Knowledge | CG | 28.38 ± 2.56 | 28.42 (26.91–29.93) | 0.001 | 0.21 |
|  | OG | 48.21 ± 2.11 | 48.15 (46.84–49.46) |  |  |
| Healthy Diet Score | CG | 24.27 ± 2.62 | 24.30 (22.75–25.85) | <0.001 | 0.18 |
|  | OG | 46.44 ± 3.14 | 46.40 (45.01–47.79) |  |  |
| Healthy Behavior Score | CG | 21.27 ± 2.52 | 21.31 (19.82–22.80) | <0.001 | 0.23 |
|  | OG | 44.60 ± 2.79 | 44.55 (43.20–45.90) |  |  |

*Adjusted for age, sex, BMI, and education level. CG: Control Group; OG: Observation Group. B Effect size (η²) interpreted as: 0.01=small, 0.06=medium, 0.14=large.

**Figure S1. CONSORT Flow Diagram of Participant Enrollment, Allocation, and Follow-up**

Excluded (n= 22)

♦  Not meeting inclusion criteria (n= 15)

♦  Declined to participate (n = 7)

Lost to follow-up (give reasons) (n= 0)

Discontinued intervention (n= 0)

Lost to follow-up (n= 0)

Discontinued intervention (n= 0)

## Follow-Up

Analysed (n= 64)

## Analysis

Analysed (n= 64)

## Enrollment

Control group (n= 64)

♦ Received routine management: (n= 64 )

♦ Analyzed: (n= 64)

## Allocation

Observation group (n= 64)

♦ Received standardized management: (n= 64)

♦ Analyzed: (n= 64)

Randomized (n= 128)

Assessed for eligibility (n= 150)
